# Anaesthetic Complications in Dogs Undergoing Total Ear Canal Ablation and Ventral Bulla Osteotomy: A Single-Centre Retrospective Study

**DOI:** 10.3390/ani15101401

**Published:** 2025-05-13

**Authors:** Anna Maria Szewczyk, Manuel Alejandro Fernandez Barrientos, Carl Bradbrook

**Affiliations:** 1 Anderson Moores Veterinary Specialists, Winchester SO21 2LL, UK; 2 Veterinary Medical Teaching Hospital, School of Veterinary Medicine, University of California Davis, Davis, CA 95616, USA

**Keywords:** TECA-BO, otitis, complications

## Abstract

Canine otitis, a prevalent and often recurrent condition in small animal veterinary medicine, can lead to severe and chronic ear disease, necessitating surgical intervention when medical management fails. In advanced cases, Total Ear Canal Ablation with Bulla Osteotomy (TECA-BO) is frequently considered the most effective treatment option to alleviate pain, resolve infection, and improve the overall quality of life of affected dogs. However, as with any major surgical procedure, TECA-BO carries inherent risks, particularly those related to anaesthesia. This single-centre retrospective study aims to evaluate and characterise anaesthesia-related complications in dogs undergoing TECA-BO surgery. The study analyses data from canine patients that underwent the procedure at a specialised veterinary referral centre over a 14-year period spanning from 2007 to 2021. The primary objective is to determine the incidence of anaesthesia-related complications and to identify any potential risk factors that may contribute to their occurrence. A statistical analysis was performed to compare the incidence of complications, with Fisher’s Exact Test being used to assess the significance of observed differences. The significance level was set at *p* = 0.05 to determine whether specific variables had a meaningful impact on anaesthesia-related complications. The findings of this study may help inform clinical decision-making, optimise anaesthetic management strategies, and ultimately improve the safety and success of TECA-BO procedures in veterinary practice.

## Simple Summary

Total Ear Canal Ablation with Bulla Osteotomy (TECA-BO) is a surgical procedure commonly performed in dogs for the management of chronic otitis media or otitis interna, particularly in cases where medical treatment has proven ineffective or when the patient’s temperament makes ongoing medical management impractical. While TECA-BO is often necessary to improve a dog’s quality of life, anaesthesia-related complications during the procedure can contribute to an increased risk of postoperative complications, potentially affecting recovery and overall surgical outcomes. This study aims to report and analyse various complications associated with TECA-BO, assess their frequency, and evaluate their impact on patient recovery. Additionally, it aims to provide valuable insights into the quality of anaesthesia care provided in a referral hospital setting, helping to identify potential areas for improvement in perioperative management.

## 1. Introduction

Morbidity and mortality during the perianaesthetic period have been extensively investigated across various animal species. Several studies have examined intraoperative anaesthetic complications in veterinary patients [1,2,3,4,5,6,7,8], particularly in procedures such as thoracolumbar hemilaminectomies [9], cardiac surgeries [10,11], and hepatic surgeries [12]. These studies have provided valuable insights into perioperative risks and outcomes. However, the incidence of anaesthetic complications associated with Total Ear Canal Ablation with Bulla Osteotomy (TECA-BO) remains unexplored.

TECA-BO is a surgical technique used in the management of ceruminous gland adenocarcinomas, extensive benign ear diseases, and failed lateral ear resections. It plays a crucial role in treating chronic otitis externa, particularly in cases where medical management is unsuccessful [13,14,15]. Despite its efficacy, TECA-BO is considered a highly invasive and painful procedure, necessitating careful evaluation of its anaesthetic implications.

This retrospective study aims to describe the anaesthetic complications encountered during TECA-BO surgeries at Anderson Moores Veterinary Specialists between 2007 and 2021 with the objective of enhancing perioperative care protocols and improving patient outcomes in TECA-BO.

## 2. Materials and Methods

### 2.1. Animals

Animals included in this retrospective study were selected by reviewing the anaesthetic records of those that underwent TECA-BO surgeries between 2007 and 2021 at Anderson Moores Veterinary Specialists. Exclusion criteria included animals that underwent TECA-BO surgery in conjunction with other surgical procedures, except for imaging studies performed under the same general anaesthesia (GA). Additional exclusion criteria included illegible, missing, or incomplete anaesthetic records.

Dogs were included in the study if their anaesthetic records contained data for heart rate (HR), mean arterial blood pressure (MAP), respiratory rate (RR), end-tidal carbon dioxide concentration (PE’CO_2_), the implementation of mechanical ventilation (MV), and temperature (T).

### 2.2. Registered Data

Recorded data included sex (F—female; FN—female neutered; M—male; MN—male neutered), breed, body weight (kg), and total GA duration (minutes). Analgesia was defined as the use of intravenous paracetamol (Y/N), the use of regional anaesthesia (Y/N), and the intraoperative use of an intravenous infusion (Y/N).

The following intraoperative complications were recorded: bradycardia, hypotension, pharmacological intervention to increase heart rate (PIHR), pharmacological intervention to treat nociception (PIN), use of intermittent positive pressure ventilation (IPPV), hypercapnia, hypothermia, haemorrhage, gastro-oesophageal regurgitation, and death. Bradycardia was defined as a heart rate of <60 beats per minute, a sudden decrease of 20% in heart rate compared to the previous value, or a heart rate of <60 beats per minute with concurrent hypotension. Hypotension was defined as a mean arterial pressure (MAP) < 60 mmHg for at least two readings taken five minutes apart over a ten-minute period. Hypercapnia was defined as PE’CO_2_ > 55 mmHg (7.3 kPa). Hypothermia was defined as a rectal or oesophageal temperature < 37 °C. Gastro-oesophageal regurgitation was recorded as the presence of visible gastric contents in the oropharynx or nares. Haemorrhage was recorded if the animal had a calculated blood loss > 10% of its blood volume. Intraoperative mortality was defined as deaths occurring between intubation and extubation.

The choice of blood pressure monitoring technique—either invasive blood pressure (IBP) or non-invasive blood pressure (NIBP)—was left to the discretion of the anaesthetist and was recorded. Information about local nerve blocks, including intraoperative splash blocks or preoperative blocks, was also collected.

### 2.3. Statistical Analysis

Data are presented as the number of cases and percentages. Fisher’s Exact Test was used to compare categorical variables, and two-sample *t*-tests were used to compare anaesthetic times between Y/N complication categories. The threshold for significance was *p* < 0.05.

## 3. Results

A total of 95 anaesthetic records were included in the study. Different demographic groups were included, as shown in Table 1. The cohort included 7 intact females (7.4%), 19 spayed females (20.0%), 30 intact males (31.6%), and 39 neutered males (41.1%). The mean was considered for inclusion in the study. The mean ± SD regarding the duration of general anaesthesia (GA), measured from induction to tracheal extubation, was 162.6 ± 50.0 min.

**Figure 1:**
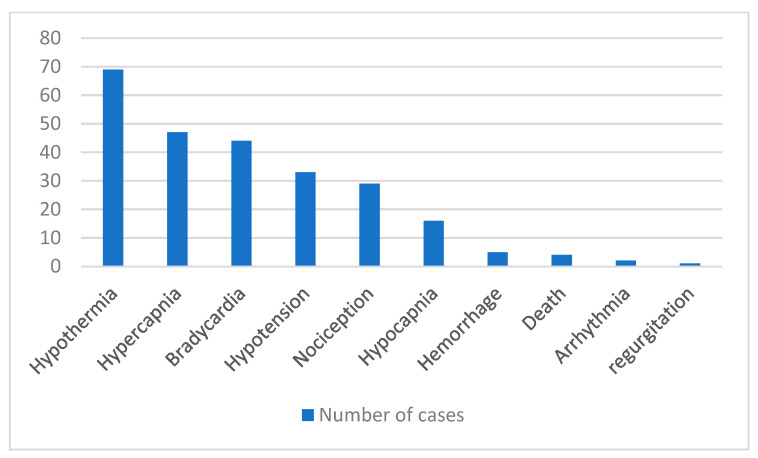
Anaesthesia and intraoperative complications.

The following anaesthetic and intraoperative complications were observed (Figure 1). Bradycardia as an anaesthetic complication was experienced in 44 (46.32%) dogs, hypotension in 33 (34.74%), hypercapnia in 47 (49.47%), hypocapnia in 16 (16.84%), hypothermia in 69 (72.63%), and arrhythmia in 2 (2.11%). One (1.05%) patient regurgitated during surgery. In 29 (30.53%) cases, pharmacological intervention to treat nociception was needed. Three (3.16%) patients received treatment for bradycardia. Intermittent positive pressure ventilation was used in 19 (20%) cases. Intraoperative haemorrhage was experienced by five (5.26%) patients. The total mortality rate for all cases was 4.21% (four cases).

## 4. Discussion

This is the first study reporting anaesthetic complications associated with TECA-BO surgery. The findings of this study suggest that patients undergoing TECA-BO surgery experience a variety of complications. Hypothermia is a common complication of general anaesthesia in canines and is associated with a prolonged recovery time [16,17]. An increased duration of anaesthesia is negatively associated with the severity of complications during TECA-BO surgery. A few studies have shown that prolonged anaesthesia has a negative impact on the occurrence of complications in different species, such as dogs, cats, and horses [18,19,20]. Another study demonstrated a significant association between the duration of surgery and failure of ambulation in dogs following hemilaminectomy for thoracolumbar intervertebral disc herniation [21].

Peri-anaesthetic hypotension has previously been associated with multiple morbidities in dogs in different types of surgeries. [22,23]. Hypotension is a common complication in anesthetised animals. In Redondo’s 2007 [3] study documenting cardiorespiratory complications, hypotension was reported in 37.9% of patients and was identified as one of the most common complications, occurring in a high percentage of cases. When compared to our results (34.74% incidence of hypotension), the findings are similar. A similar percentage of hypotension was observed in dogs undergoing thoracolumbar hemilaminectomy in Bruniges’ [9] study (33.9%).

Significant haemorrhage during surgery is a rare complication, with several studies showing that patients may need blood transfusions [24,25]. The main sources of bleeding during TECA are the retroarticular vein, the external carotid artery, the maxillary vein, and the internal carotid artery [26]. Haemorrhage may be difficult to manage due to difficulty accessing the bleeding vessel, which may explain the higher mortality rate. Minimising trauma and ensuring that a surgeon with greater experience performs the dissection are both likely to decrease the risk of haemorrhage [27].

In our study, the mortality rate was 4.2%, which is notably higher than rates reported in the literature. Anaesthesia-related mortality in dogs has been estimated at approximately 0.05% in healthy populations and 1.33% in sick populations, with an overall rate of 0.17% [2]. A more recent study reported a mortality rate of 0.69% in dogs based on a large global dataset of over 55,000 canine anaesthetic procedures [28].

In this study, the incidence of haemorrhage as a complication and the mortality rate were similar, which the authors postulate may account for the number of deaths observed. The sample size is low compared to other mortality studies, which is one of the limitations of our study.

### Limitations

Several limitations in the present study must be highlighted. The data were collated and analysed retrospectively in a single referral centre. Additionally, the number of cases included in the study is a limitation. The data did not compare different anaesthetic protocols, which may have had an impact on the reported complications. Data on the co-occurrence of multiple complications in individual dogs were not collected.

## 5. Conclusions

This study provides valuable insights into the anaesthetic complications associated with Total Ear Canal Ablation with Bulla Osteotomy (TECA-BO) in dogs. Our findings indicate that hypothermia, hypercapnia, and hypotension are the most frequently encountered anaesthetic complications, with hypothermia being the most prevalent. The overall mortality rate observed (4.2%) was higher than that reported in other studies, which may be influenced by the small sample size and the severity of the cases included. Additionally, haemorrhage, although infrequent, was noted in a subset of cases and could have contributed to perioperative mortality.

While this study does not evaluate specific anaesthetic protocols or interventions, it helps to establish a clearer understanding of the frequency and types of complications that may arise during TECA-BO procedures. This information can support the development of future studies aimed at identifying risk factors and assessing the effectiveness of targeted perioperative management strategies. Ultimately, recognising the common complications observed in this context may encourage more vigilant monitoring and informed clinical decision-making, indirectly contributing to improved patient safety and outcomes in veterinary practice.

## Figures and Tables

**Table 1:** This table presents the breed distribution and corresponding percentages of dogs included in the study.

Number Per Breed	Breeds	Total Count	Percent (%)
1	Australian Terrier, Beagle, Boxer, Bulldog, Cockapoo, English Setter, Flat-Coated Retriever, Fox Terrier, Labradoodle, Maltese Terrier, Poodle, Red Setter, Siberian Husky, Spinoni, Springer Spaniel, Swedish Valhund, Wheaten Terrier, Whippet	19	19.95
2	American Cocker Spaniel, Border Collie, German Shepherd, Jack Russell Terrier, Pug, St. Bernard	12	12.63
3	Border Terrier, Cavalier King Charles Spaniel, French Bulldog, Rottweiler, Sharpei, Shih-Tzu, Staffordshire Bull Terrier, Welsh Terrier	24	25.26
5	Golden Retriever	5	5.26
6	Crossbreed	6	6.32
8	Labrador	8	8.42
10	West Highland White	10	10.53
11	Cocker Spaniel	11	11.58

## Data Availability

The raw data supporting the conclusions of this article will be made available by the authors upon request.

## Abbreviations

The following abbreviations are used in this manuscript:

TECA-BO	Total ear canal ablation with bulla osteotomy
GA	General anaesthesia
HR	Heart rate
MAP	Mean arterial pressure
RR	Respiratory rate
PE’CO_2_	End-tidal carbon dioxide concentration
MV	Mechanical ventilation
T	Temperature
F	Female
FN	Female neutered
M	Male
MN	Male neutered
PIHR	Pharmacological interaction to increase heart rate
PIN	Pharmacological intervention to treat nociception
IPPV	Intermittent positive pressure ventilation
IBP	Invasive blood pressure
NIBP	Non-invasive blood pressure
